# Gene expression signatures associated with chronic endometritis revealed by RNA sequencing

**DOI:** 10.3389/fmed.2023.1185284

**Published:** 2023-07-20

**Authors:** Kyoko Oshina, Keiji Kuroda, Kazuhiko Nakabayashi, Junko Tomikawa, Mari Kitade, Rikikazu Sugiyama, Kenichiro Hata, Atsuo Itakura

**Affiliations:** ^1^Department of Maternal-Fetal Biology, National Center for Child Health and Development, Setagaya, Tokyo, Japan; ^2^Department of Obstetrics and Gynecology, Juntendo University Faculty of Medicine, Bunkyo, Tokyo, Japan; ^3^Center for Reproductive Medicine and Endoscopy, Sugiyama Clinic Marunouchi, Chiyoda, Tokyo, Japan; ^4^Center for Reproductive Medicine and Implantation Research, Sugiyama Clinic Shinjuku, Shinjuku, Tokyo, Japan; ^5^Department of Human Molecular Genetics, Gunma University Graduate School of Medicine, Maebashi, Gunma, Japan

**Keywords:** chronic endometritis, endometrium, menstrual cycle, RNA sequencing, differentially expressed genes

## Abstract

**Introduction:**

Chronic endometritis (CE) is a persistent inflammatory condition of the endometrium characterized by the infiltration of plasma cells in the endometrial stroma. CD138 immunohistochemistry is considered to improve the CE diagnosis rate.

**Methods:**

Using the number of CD138-positive cells equal or greater than five as a diagnostic criterion for CE, we identified 24 CE and 33 non-CE cases among women with infertility. We conducted RNA-sequencing analysis for these 57 cases in total as an attempt to elucidate the molecular pathogenesis of CE and to search for new biomarkers for CE.

**Results and Discussion:**

By comparing CE and non-CE groups, we identified 20 genes upregulated in the endometria of CE patients, including 12 immunoglobulin-related genes and eight non-immunoglobulin genes as differentially expressed genes. The eight genes were *MUC5AC*, *LTF*, *CAPN9*, *MESP1*, *ACSM1*, *TVP23A*, *ALOX15*, and *MZB1*. By analyzing samples in the proliferative and secretory phases of the menstrual cycle separately, we also identified four additional non-immunoglobulin genes upregulated in CE endometria: *CCDC13* by comparing the samples in the proliferative phase, and *OVGP1*, *MTUS2*, and *CLIC6* by comparing the samples in the secretory phase. Although the genes upregulated in CE may serve as novel diagnostic markers of CE, many of them were upregulated only in a limited number of CE cases showing an extremely high number of CD138-positive cells near or over one hundred. Exceptionally, *TVP23A* was upregulated in the majority of CE cases regardless of the number of *CD138*-positive cells. The upregulation of *TVP23A* in the endometria of CE cases may reflect the pathophysiology of a cell-type or cell-types intrinsic to the endometrium rather than the accumulation of plasma cells. Our data, consisting of clinical and transcriptomic information for CE and non-CE cases, helped us identify gene expression signatures associated with CE.

## 1. Introduction

Chronic endometritis (CE) is a persistent inflammatory disease of the uterine endometrium. Intrauterine infection is considered to be the main cause. Chronic endometritis is generally asymptomatic, but is strongly associated with implantation failure and miscarriage (1–3). Therefore, accurate diagnosis and treatment of CE are considered to be critical for improving the pregnancy rate and shortening the time to pregnancy in subsequent fertility treatments. Large-scale prospective cohort studies are awaited to demonstrate the utility of treatment benefit on clinical outcome for CE.

Chronic endometritis is histologically defined as plasma cell infiltration of the endometrial stroma. It is important to diagnose it using a clinical test based on a highly sensitive molecular marker, in addition to regular histological examination. The endometrium abundantly contains immunocompetent cells, such as natural killer (NKs) cells, macrophages, T cells, and neutrophils, but rarely contains B-lymphocytes and plasma cells (4–7). However, in the endometrium of women with CE, B-lymphocytes and plasma cells have been reported to be present (7, 8). Further, CD138 is a transmembrane heparin sulfate proteoglycan that acts as an extracellular matrix receptor and is involved in several cellular functions (9, 10). Additionally, CD138 immunostaining has been shown to be reliable in the detection of plasma cells in the evaluation and classification of hematologic malignant neoplasms, such as B-cell lymphoma (11). Therefore, CD138 immunohistochemistry staining of endometrial tissues was applied to the diagnosis of CE (12–14). Although the diagnostic accuracy of CD138 immunostaining has been reported to be high in a number of studies (14–16), the results tend to be inconsistent among studies, largely because of the lack of unified diagnostic criteria for CE (e.g., the criterion for the number of CD138-positive cells) (17, 18). Although CD138 immunohistochemistry was once considered to improve the CE diagnosis rate, it has remained debatable whether CD138 is a marker of infection-induced inflammation in the endometrium (19, 20).

The mechanisms by which CE contributes to poor pregnancy outcomes have yet to be elucidated. In this study, we obtained transcriptome data of endometrial tissues from 24CE cases and 33 non-CE cases by RNA sequencing and searched for genes differentially expressed in CE cases. Our data, consisting of clinical and transcriptomic information for CE and non-CE cases, helped us identify gene expression signatures of the immunoglobulin-related and the non-immunoglobulin genes associated with CE.

## 2. Patients and methods

### 2.1. Patients

This study was approved by the Ethics Committees of Juntendo University Medical Hospital, Sugiyama Clinic, and the National Center for Child Health and Development. Written consent was obtained from women with infertility who visited the Sugiyama Clinic (Shinjuku, Tokyo, Japan) for CE examinations. Patients with a history of antibiotics within 3 months were excluded. Endometrial tissue was collected from 190 patients between July 2020 and April 2021. All the patients were healthy. Endometrial biopsy was performed on an outpatient basis. CE was diagnosed using CD138 immunohistochemistry staining. Using G*Power (21), we estimated the sample size required for 80% power to detect differentially expressed genes in CE as 60 (30 cases and 30 controls). The prevalence of CE among the infertility patients who visited Sugiyama Clinic, used in the power calculation, was 40% (odds ratio 4.5). We then randomly selected 35 each of CE and non-CE cases, and subjected them to RNA sequencing analysis to obtain data of at least 30 each of CE and non-CE cases. After obtaining RNA sequencing data, we found that some CE and non-CE individuals received hormone therapy before endometrial biopsy. After excluding those individuals, the numbers of CE and non-CE ended up with 24 and 33 cases, respectively (Supplementary Tables S1, S2). The patient characteristics for 24 CE and 33 non-CE cases that were subjected to RNA-seq analysis are summarized in Table 1.

**Table 1:** Comparison of patients’ characteristics between the CE (*n* = 24) and non-CE (*n* = 33) groups.

Item	CE (*n* = 24)	Non-CE (*n* = 33)	Value of *p*
Age: average [SD]	39.08 [3.74]	35.45 [4.72]	0.010
Gravidity experience (%)	11/24 (45.8)	12/33 (36.4)	0.587
Pregnancy experience (%)	4/24 (16.7)	3/33 (9.1)	0.439
Number of embryos transferred: average [SD]	2.63 [3.78]	3.03 [2.61]	0.189
Repeated implantation failure (%)	7/24 (29.2)	17/33 (51.5)	0.110
Recurrent pregnancy loss (%)	2/24 (8.3)	2/33 (6.1)	1.000
Length of Infertility (year): average [SD]	3.61 [2.76]	2.64 [1.54]	0.593
Anti-Mullerian Hormone (ng/ml): average [SD]	3.40 [3.92]	3.61 [2.48]	0.337
Endometriosis (%)	3/24 (12.5)	4/33 (12.1)	1.000
Detection of intrauterine bacteria other than Lactobacillus (%)	4/24 (16.7)	2/33 (6.1)	0.228
Presence of endometrial polyps (%)	14/24 (58.3)	10/33 (30.3)	0.057
CD 138 count: average [SD]	55.0 [81.0]	1.24 [1.37]	–

For all clinical items, individual data are available in the Supplementary Table S1.

### 2.2. Statistical analysis of the patients’ clinical information

Statistical analyses of patients’ clinical information were conducted in the R environment (version 4.2.1). Thirteen items of the patients’ clinical information are presented as a mean with a standard deviation for CE (*n* = 24) and non-CE (*n* = 33) groups. Categorical and numerical variables were assessed to determine statistical significance of the difference between the two groups using Fisher’s exact test and Mann–Whitney *U*-test, respectively, using R with a significance threshold *p-*value of 0.05.

### 2.3. Endometrial biopsy

Endometrial tissues were collected using an endometrial curette (Pipet Curet, Fuji Medical Corporation) after the vagina was washed with saline and divided into two aliquots. One aliquot was placed in 10% neutral buffered formalin for subsequent immunostaining. The other aliquot was placed in RNAlater (Qiagen) and temporarily stored at 4°C for a few days or at –80°C for a longer period when not immediately subjected to RNA isolation.

### 2.4. Immunohistochemistry staining and diagnostic criterion of CE

Formalin-fixed endometrial tissues were sent to BML, Inc. (Tokyo, Japan) for CD138 immunohistochemical staining using anti-CD138 antibodies (M7228, Dako). The tissues were kept in formalin prior to paraffin embedding usually for less than 24 h and occasionally for 48–72 h. Pathologists counted the number of CD138-positive plasma cells per microscopic view field at 400-fold magnification for 10 random fields. The patients were classified into the CE or non-CE group, depending on whether the total number of CD138-positive cells in 10 fields was ≥5 (15, 22).

### 2.5. Total RNA extraction

Endometrial tissues treated with RNAlater were thawed at room temperature and frozen. The blood clots adhering to the tissue were removed under a microscope. Total RNA was extracted using the AllPrep DNA/RNA/miRNA Universal Kit (80,224, QIAGEN), according to the manufacturer’s protocol. The tissue was lysed with 600 μl of Buffer RLT plus using an 18-gauge needle. The RNA concentration was measured using a Nanodrop 8,000 spectrophotometer (Thermo Scientific). The RNA was electrophoresed using an RNA Pico 6,000 kit and an Agilent 2,100 Bioanalyzer (Agilent Technologies) to check its quality. An RNA Integrity Number (RIN) between 1 and 10 was used to estimate RNA quality, and RNA samples with a RIN >7 were used for RNA sequencing.

### 2.6. RNA sequencing (RNA-seq)

RNA-sequencing libraries were prepared using the NEBNext rRNA Depletion Kit v2 (E7405L, NEB) and NEBNext Ultra II Directional RNA Library Prep Kit (E7775S, NEB), according to the manufacturer’s protocols with 1 μg of total RNA as starting material and with seven polymerase chain reaction cycles for library amplification. The RNA-seq libraries were sequenced on a HiSeq X platform (Illumina) at Macrogen Japan Corp. (Tokyo, Japan). Sequence reads in FASTQ format were aligned to a *Homo sapiens* transcript fasta set using a reference genome-free method, Salmon (23). The number of genes in the GTF file (Homo_sapiens.GRCh37.69.gtf) used was 60,230. The read counts of all the samples obtained by Salmon were combined into a single data frame using tximport (24) and further analyzed using EdgeR (25). The count data were converted to counts per gene per million mapped reads (CPM). After removing low-count data (CPM <1), raw read counts were normalized using the trimmed mean of *M*-values (TMM) normalization. Pearson’s correlation coefficients of gene expression profiles were measured between samples to determine differences in gene expression profiles among the groups. Differentially expressed genes (DEGs) were selected based on value of *p*s and false discovery rates (FDR). Gene expression levels transformed into *Z*-scores were visualized as heatmaps using the R package *Complexheatmap*. The weighted gene co-expression network analysis tool (26) was used for hierarchical clustering to identify the modules. To measure the association between modules and phenotypic traits, clustering analysis was performed again with additional clinical information for each sample.

### 2.7. Quantitative reverse-transcription PCR

One microgram of total RNA was subjected to first-strand cDNA synthesis using PrimeScript™ RT reagent Kit with gDNA Eraser (Takara Bio RR047A). Real-time PCR reactions were performed with technical duplicates on the 7,500 Fast Real-Time PCR System (Applied Biosystems, Foster City, CA, USA) using TB Green® Premix Ex Taq™ II (Tli RNaseH Plus) (Takara Bio RR820A) according to the manufacturer’s instructions. PCR primers used were 5′-GCAACAGTGACATTGGCAAG-3′ and 5′-GAATCTCCAGCCCCTCGAG-3′ for *TVP23A*, and 5′-CCAACTGGGACGACATGG-3′ and 5′-GGGCACAGTGTGGGTGAC-3′ for *ACTB*. One fortieth amount of cDNA was used for one real-time PCR reaction. Relative expression levels of *TVP23A* were calculated by the ΔΔCt method using the Ct values of *ACTB* as a reference gene. Expression levels relative to the median expression level among non-CE cases were determined for all samples and subjected to Mann–Whitney U test to assess difference between CE and non-CE groups.

### 2.8. Immune cell analysis

The proportions of 22 types of immune cells in each endometrial tissue (CE and non-CE) were estimated by analyzing RNA sequencing data using the CIBERSORT algorithm (27). The estimated proportions of infiltrated immune cells were compared between the CE and non-CE groups using a *t-test* with a statistical significance threshold *p-*value of 0.05.

## 3. Results

### 3.1. Sample selection, CE diagnosis, and classification by endometrial cycle days

**Figure 1:**
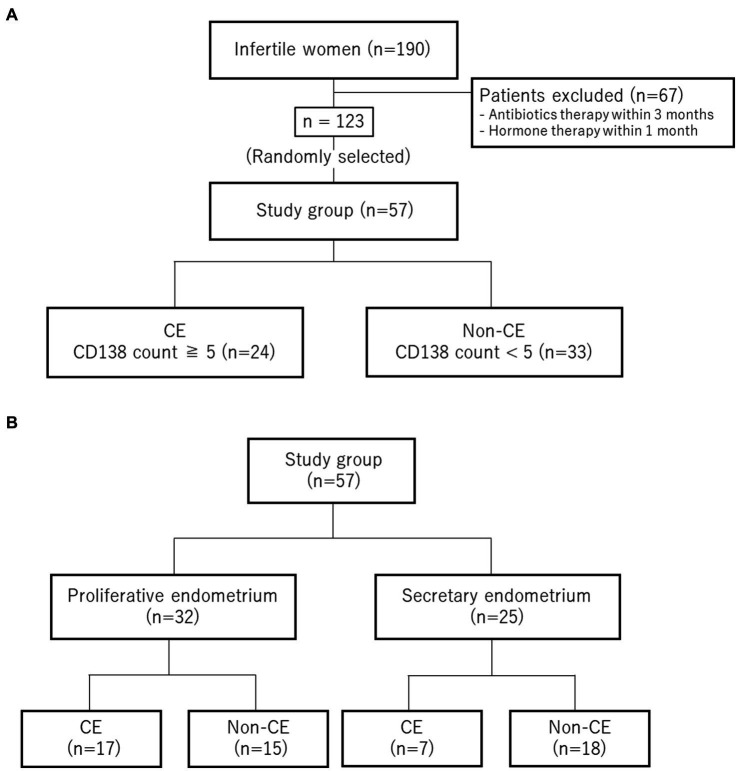
Sample classification. **(A)** Selection and diagnosis for CE. **(B)** Numbers of the cases in the proliferative phase and in the secretory phase.

After excluding 67 patients who received antibiotic therapy within the past 3 months or hormone therapy within the past month of endometrial tissue collection, 57 of 123 patients were randomly selected as participants of this study (Figure 1A). Chronic endometritis was diagnosed on the basis of CD138 immunohistochemical staining. Twenty-four patients were diagnosed with CE (CD138 count ≥5). Thirty-three patients were not positive (CD138 counts <5) and were categorized as non-CE (Figure 1A). Endometrial samples were collected during either the proliferative phase (defined as 0–14 days after the last menstrual period) or the secretory phase (defined as 15 days or later) from one patient. The CE group (*n* = 24) consisted of 17 patients in the proliferative phase and 7 in the secretory phase, whereas the non-CE group (*n* = 33) consisted of 15 patients in the proliferative phase and 18 in the secretory phase (Figure 1B).

### 3.2. Identification of genes differentially expressed between CE and non-CE groups

**Figure 2:**
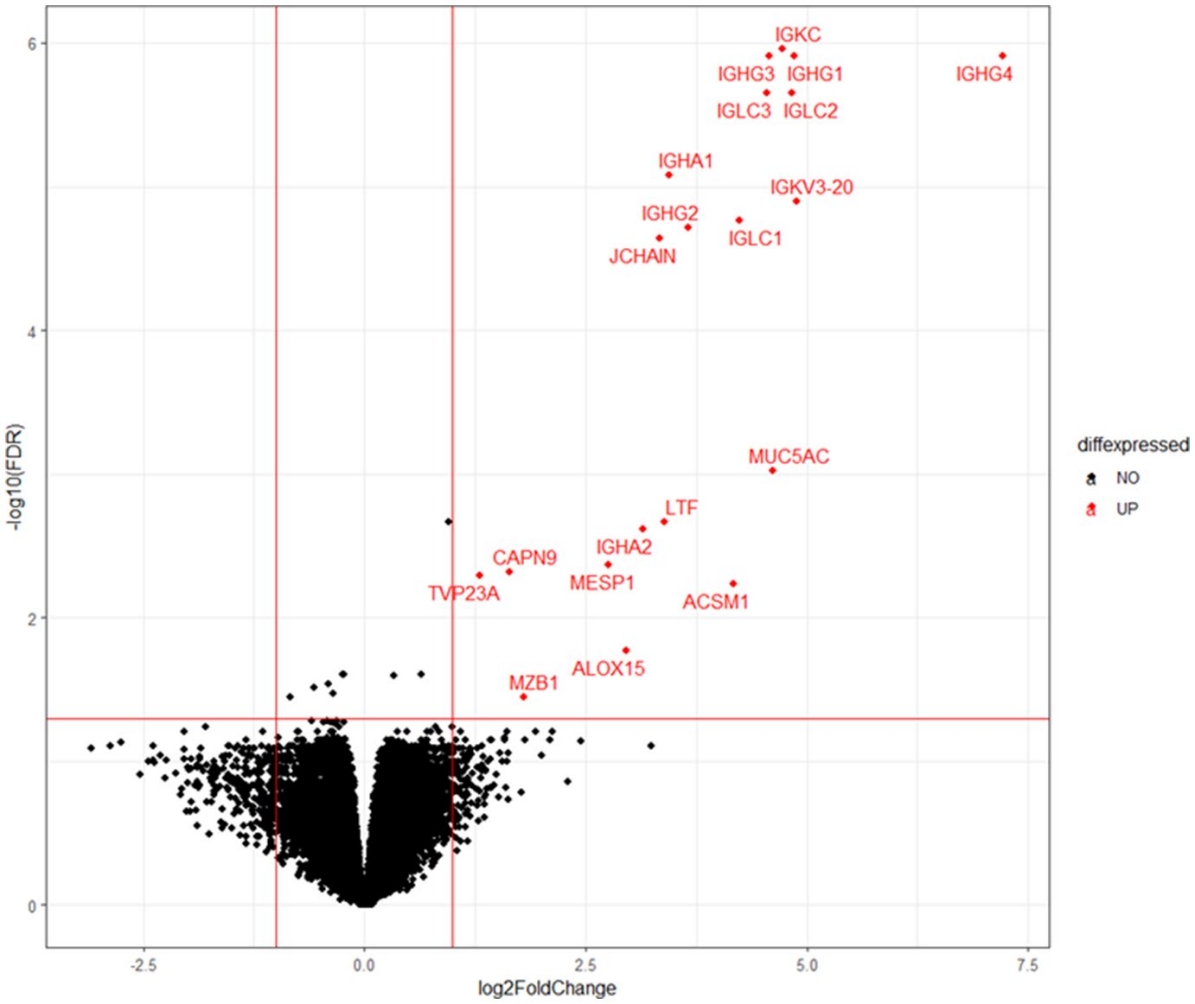
Volcano plot representation of DEGs. *X*-axis represents log_2_ values of the fold change of gene expression in the CE group compared to the non-CE group, and the red vertical lines indicate threshold values of log2-transformed fold change (<−1 or >1) for DEG selection. *Y*-axis represents the –log_10_ value of FDR *q* value, and the red horizontal line indicates the threshold value (FDR < 0.05). Twenty genes selected as DEGs are shown by red dots.

**Figure 3:**
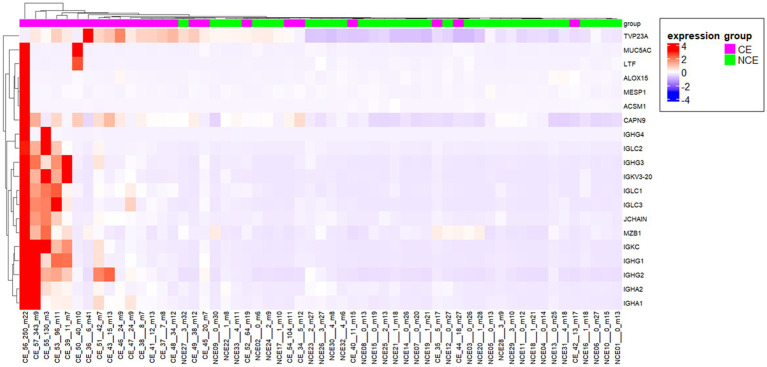
Heatmap representation of the 20 genes upregulated in CE endmetria. The sample names below the heatmap include information of the sample type [CE or non-CE (NCE)], the number of CD138-positive cells (ranging from 0 to 343), and days after the last menstrual period (ranging from m3 to m41).

**Figure 4:**
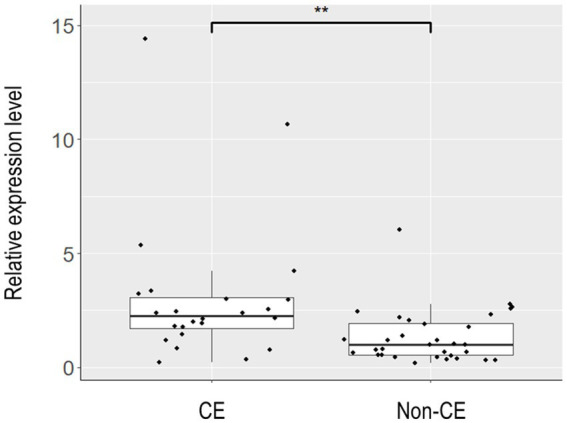
Box plot representation of the relative expression levels of TVP23A in endometrial tissues. Endometrial tissues of 33 non-CE cases and 24 CE cases were subjected to quantitative reverse-transcription PCR to assess the relative expression levels of TVP23A. After excluding one non-CE sample that showed extremely poor PCR amplification for ACTB, 32 non-CE cases and 24 CE cases were subjected to the calculation of the relative expression levels of TVP23A and the subsequent Mann–Whitney *U* test. The expression level of TVP23A was statistically significantly higher in the CE group than in non-CE group (**p = 0.0014).

To elucidate the physiology of CE at the molecular level, we conducted mRNA sequencing analysis of the endometrial tissues of 33 CE and 24 non-CE patients. Approximately 20 million read pairs were obtained from all the 57 samples. After removing genes with low mapped read counts, 18,257 genes were subjected to TMM normalization and subsequent analyses. In a differential gene expression analysis between the two groups with the selection criteria of FDR <0.05, and log2-transformed fold change value <1, or >1, 20 upregulated genes and no downregulated genes in the CE group compared to the non-CE group were identified (Figure 2 and Supplementary Table S1). The top 11 upregulated genes in the CE group, *IGKC*, *IGHG1*, *IGHG4*, *IGLC3*, *IGHG3*, *IGLC2, IGHA1*, *IGKV3-20*, *IGLC1*, *IGHG2*, and *JCHAIN* (Figure 2), as well as the 14th gene, *IGHA2*, were all related to immunoglobulins, and their expression levels were proportional to the number of CD138-positive cells in endometrial tissues (Figure 3). Among the remaining nine non-immunoglobulin genes, all but *TVP23A* were highly expressed in one or two CE samples such as “CE_56_250_m22” and “CE_57_343_m9,” whose numbers of CD138-positive cells were extremely high (Figure 3). Only *TVP23A* consistently showed elevated gene expression in the CE group samples, regardless of the number of CD138-positive cells, ranging between 5 and 343 (Figure 3 and Supplementary Data S1). The elevated gene expression of *TVP23A* in the CE group was verified by a quantitative PCR analysis for the CE group (*n* = 24) and the non-CE group (*n* = 33) samples (Figure 4).

### 3.3. Identification of DEGs between the CE and non-CE groups during the proliferative and secretory phases

**Figure 5:**
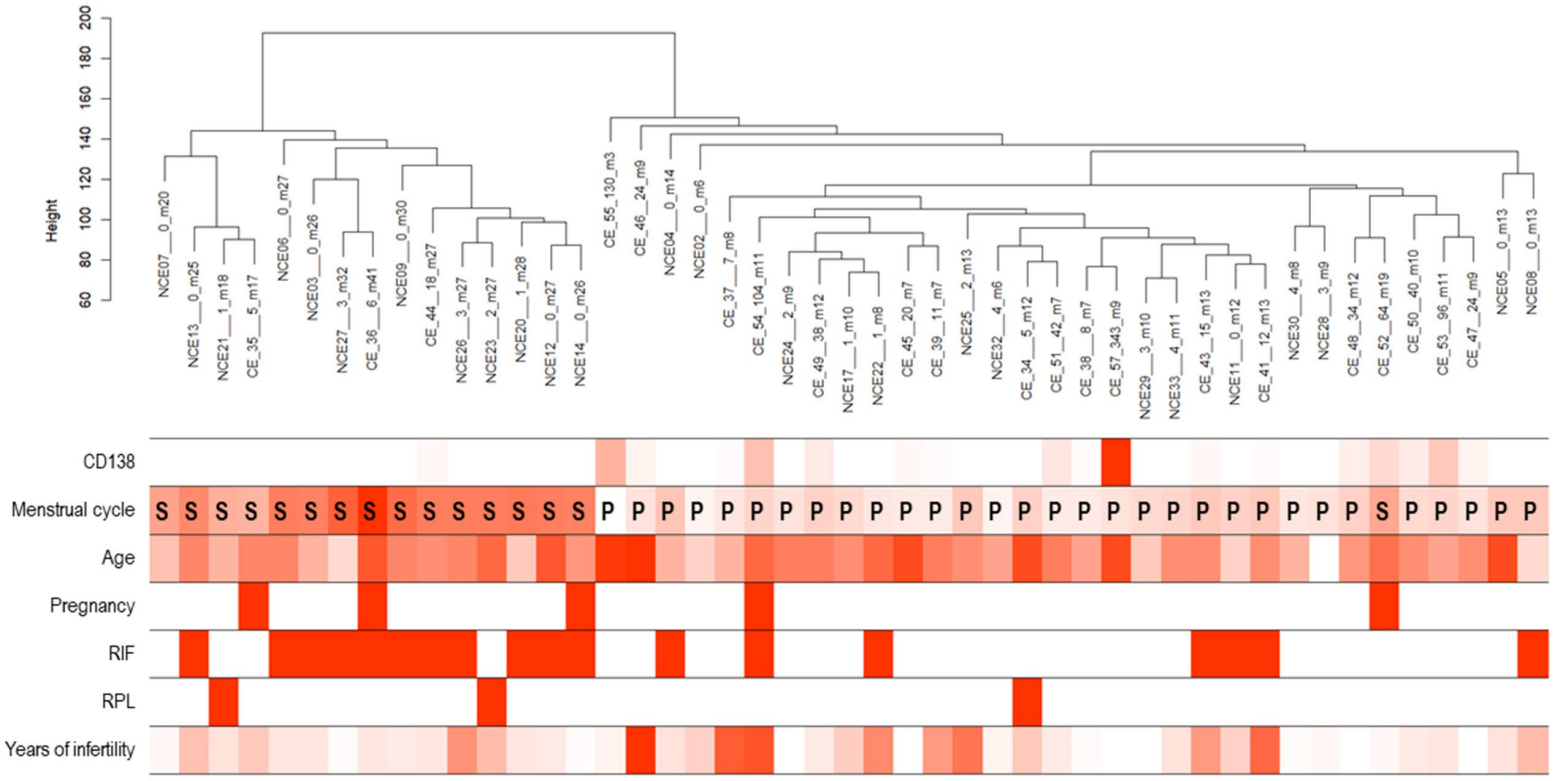
**(Top)** A dendrogram generated by the hierarchical clustering of CE and non-CE samples with a cut-off threshold of 170 (height). **(Bottom)** Seven clinical features. Color intensity of each row is proportional to the followings: number of CD138-positive cells, days after the last menstrual period, age, times of past pregnancies, presence or absence of recurrent implantation failure (RIF), presence or absence of recurrent pregnancy loss (RPL), and infertility years.

**Figure 6:**
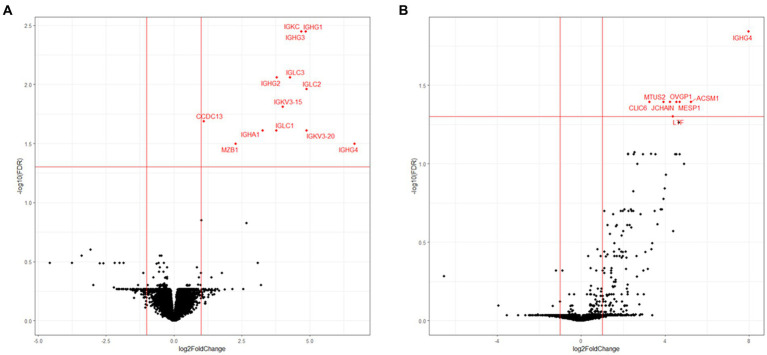
Volcano plot representation of DEGs between the CE and non-CE group samples in the proliferative phase **(A)** and secretory phase **(B)**. See the legend for Figure 2 for the definitions of *X* and *Y*-axes, and red lines.

Principal component analysis for all samples and genes showed no obvious separation between the CE and non-CE groups (data not shown). In an unsupervised clustering analysis for the dataset of 46 selected samples, the samples were divided into two clusters, both of which were mixed with CE and non-CE samples (Figure 5, top). By aligning seven clinical features with the clustering tree (Figure 5, bottom), we confirmed that two clusters corresponded to 15 samples in the secretory phase (the left cluster) and 31 samples in the proliferative phase. The 11 samples removed by the cutoff applied (<170 in height) were not clustered into these two major clusters. Therefore, we compared DEGs between the CE and non-CE groups in each of the two phases. In the comparison of 17 CE samples and 15 non-CE samples in the proliferative phase, 13 genes were upregulated in the CE group: 11 immunoglobulin genes, *CCDC13*, and *MZB1* (Figure 6A). In the comparison of seven CE samples and 18 non-CE samples in the secretory phase, eight genes were upregulated in the CE group: two immunoglobulin genes (*IGHG4* and *JCHAIN*) and six non-immunoglobulin genes (*OVGP1*, *MTUS2*, *CLIC6*, *ACSM1*, *MESP1*, and *LTF*) (Figure 6B). The four underlined genes were not identified as DEGs when samples from both phases were analyzed together.

### 3.4. CIBERSORTx analysis to profile immune cells in the endometrium of CE patients

**Figure 7:**
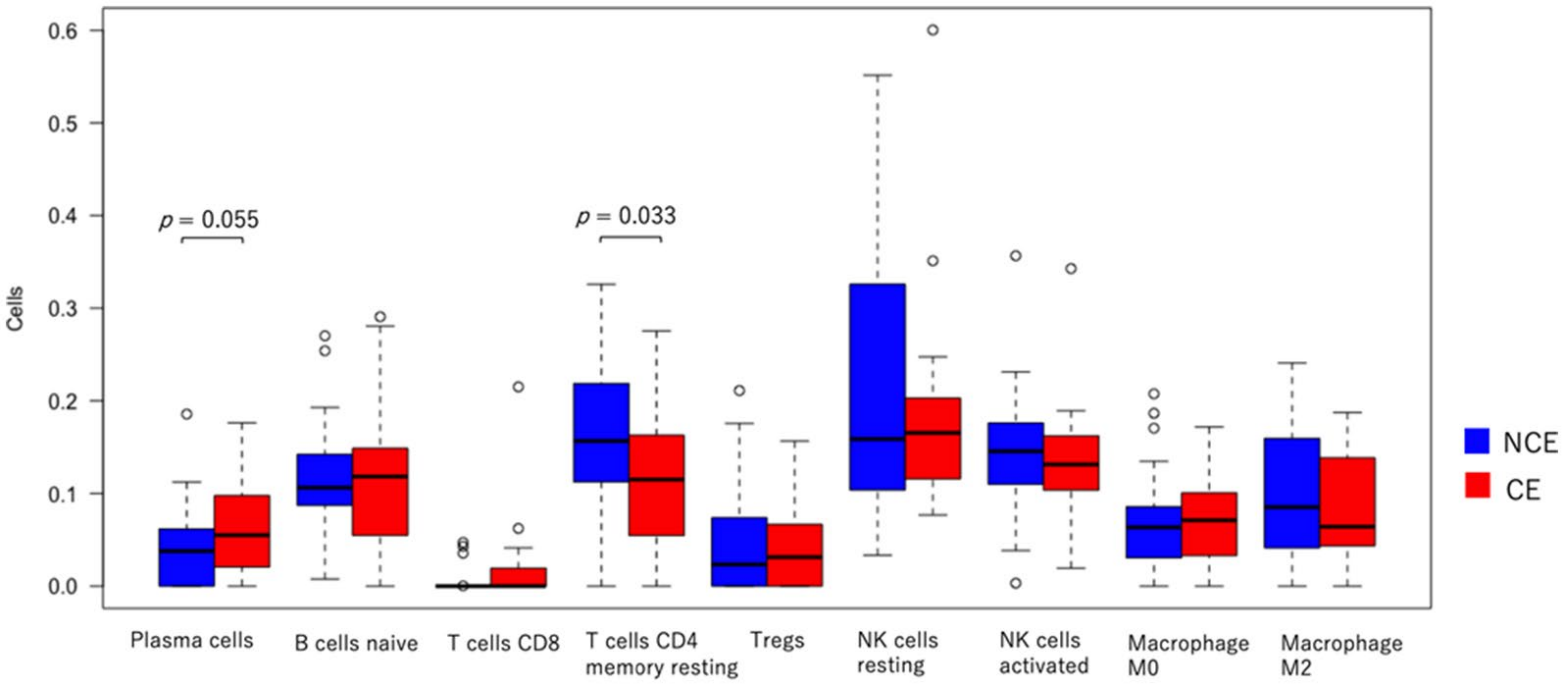
Comparison of abundance of immune cells in the CE (red) and non-CE (blue) endometria estimated by CIBERSORTx. The results of nine representative cell types among 22 types are shown.

We assessed whether any immune cells other than plasma cells increased their residential cell number in the endometrium of CE patients compared to that in non-CE patients. CIBERSORTx is an analytical tool for imputing gene expression profiles, and it provides an estimation of the abundance of member cell types in a mixed cell population using gene expression data. It uses 547 immune cell-related gene expression values to estimate the abundance of 22 types of immune cells in the sample of interest (28). By subjecting our transcriptome data of CE and non-CE endometria to CIBERSORTx, we were able to estimate the abundance of nine types of immune cells in CE and non-CE samples (Supplementary Table S3). Consistent with the diagnosis of CE using the plasma cell marker CD138, plasma cells tended to be more abundant in the CE samples than in the non-CE samples, although the *t-test* value of *p* (0.055) did not reach a statistically significant threshold (0.05) (Figure 7). However, CD4 memory T cells (resting) were less abundant in the CE samples than in the non-CE samples with statistical significance (*p* = 0.033) (Figure 7).

## 4. Discussion

### 4.1. Detection of immunoglobulin and non-immunoglobulin genes up-regulated in CE endometria

We discovered genes whose expression levels were elevated in CE endometria compared to non-CE endometria: 13 immunoglobulin-related genes and 12 non-immunoglobulin genes. It has been reported that plasma cells are more likely to be present during the proliferative phase compared with the secretory phase (14, 29–31). However, in this study, the numbers of CD138-positive cells in the proliferative phase (56.06 +/− 80.18 s.d; *n* = 17) and those in the secretory phase (52.43 +/− 82.83 s.d.; *n* = 7) were not significantly different among 24CE cases. B cells, including plasma cells, are a minor cell population in the endometrium of both proliferative and secretory phases. The numbers of B cells did not change significantly between two phases when the results of the previous flow cytometry studies were reviewed (7). In this study, the ratios of immunoglobulin-related genes and non-immunoglobulin genes among the DEGs identified were strikingly different between two phases. Whereas 11 out of 13 genes (84.6%) upregulated in the CE endometria were immunoglobulin-related genes in the proliferative phase (Figure 6A), only two out of eight genes (25%) upregulated in CE endometria were immunoglobulin-related genes (*IGHG4* and *JCHAIN*) in the secretory phase (Figure 6B). It remains to be determined in what cell-types the 12 non-immunoglobulin genes up-regulated in CE endometria are expressed. Single-cell RNA sequencing (scRNA-seq) analysis is a promising approach to further delineate gene expression signatures associated with CE in each of the cell-types in the endometrium including plasma cells and other minor immune-cell types in each of the proliferative and secretory phases.

Consistent with the primary characteristic of CE, plasma cell infiltration, 13 immunoglobulin-related genes were identified as genes upregulated in the CE endometria. However, although bacterial infection is considered to be the major trigger of CE, genes such as inflammatory cytokine genes were not detected as DEGs in this study. This is inconsistent with a previous report (32), which conducted an RNA sequencing analysis for the endometria of 32 CE patients and 72 non-CE patients. Chen and the colleagues identified 93 DEGs, and found that these DEGs were enriched in immune and inflammatory-related gene ontology terms (32). This inconsistency may be explained by the difference in the diagnostic criteria for CE between two studies. However, because the detailed clinical information as well as the diagnostic criteria were not available from their report (32), we did not pursue on this issue any further. Another relevant issue to be considered is that many of the DEGs identified in our study were highly expressed in a limited number of CE endometria whose number of CD138 positive cells was extremely high (Supplementary Table S1 and Figure 3). Taken together, the results from this and Chen’ studies indicate the necessity of additional transcriptome data for larger numbers of CE and non-CE endometria accompanied with detailed clinical information to further understand the molecular pathophysiology of CE.

One limitation of our study is that RNA sequencing data and the CD138 immuno-staining data were obtained not for the same cell populations. A piece of endometrial tissue was divided into two halves. One half was subjected to CD138 immuno-staining, and the other to RNA sequencing. Even though two halves were adjacently located, the numbers of CD138-positive cells may have been unignorably different due to the locally fluctuated nature of plasma cells. Such a difference if any may have affected gene expression data more remarkably in the cases whose number of CD138-positive cells was relatively low (e.g., zero to five). In addition to the above-mentioned scRNA-seq analysis, spatial gene expression techniques are expected to be useful to verify the priority of considering three-dimensional cellular contexts in the gene expression analysis of CE.

### 4.2. Identification of the TVP23A gene as a novel class of CE-specific marker

While most consider the plasma cell as necessary for the diagnosis of CE, some have questioned this notion (19). Therefore, if CE-specific gene expression markers are identified, they will serve as novel diagnostic markers of CE. However, many of such genes identified in this study were upregulated only in a limited number of CE cases showing an extremely high number of CD138-positive cells near or over one hundred. However, *TVP23A* was upregulated in the majority of CE cases regardless of the number of CD138-positive cells (Figure 3). The upregulation of *TVP23A* in the endometria of CE cases may reflect the pathophysiology of a cell-type or cell-types intrinsic to the endometrium rather than the accumulation of plasma cells.

*TVP23A* encodes the trans-Golgi network vesicle protein 23 homolog A (formerly known as *FAM18A*). Although *TVP23A* has recently been reported as a candidate gene for late-onset Parkinson’s disease (33), it has not been described in endometrium-related literature. According to the Human Protein Atlas^1^ (34), *TVP23A* was mostly highly expressed in macrophages among the 29 cell-types identified by the analysis of scRNA-seq data for the normal endometrium [NCBI/GEO accession number GSE111976; (35)]. The proportion of uterine CD68+ macrophages has been shown to be elevated in CE patients (36). However, it is unknown whether *TVP23A* is most highly expressed in the macrophages in the endometrium of the CE patients. *TVP23A* may be highly expressed in plasma cells because plasma cells actively secreting immunoglobulins are known to have large and highly developed Golgi apparatuses (37, 38). scRNA-seq data obtained from the endometria of CE patients will help clarifying in which cell-type(s) *TVP23A* is highly expressed and whether *TVP23A* play a critical role in the pathophysiology of CE. Functional characterization of TVP23A is required to establish the expression level of the *TVP23A* gene as a new diagnostic criterion of CE.

### 4.3. Alteration of immune cell populations in the CE endometria

The CIBERSORTx analysis for our CE transcriptome data revealed an increased tendency of plasma cells and a decreased tendency of CD4 memory T cells in the CE endometria compared to the non-CE endometria (Figure 7). In chronic infections and cancers, continued antigen persistence impedes proper adaptive immune responses, compromising the proper formation of the memory T-cell population (39). Therefore, we postulate that memory CD4 T cells tend to decrease in CE endometria because of chronic inflammation in the tissues.

## 5. Conclusion

We discovered 13 immunoglobulin-related genes and 12 non-immunoglobulin genes whose expression levels were elevated in CE. All immunoglobulin-related genes and the majority of non-immunoglobulin genes were identified as DEGs in CE likely due to the existence of plasma cells but not because of gene expression changes in other major cell types in the endometrium. However, since *TVP23A* was exceptionally found to be upregulated in the majority of CE cases regardless of the number of CD138-positive cells, this gene represents a candidate marker useful for the development of a novel molecular diagnostic method for CE. RNA sequencing is an effective approach for further understanding of the molecular physiology of CE. Our RNA sequencing data together with detail clinical information serve as a basis for future researches towards elucidating possible mechanisms through which CE involves reproductive failure.

## Data availability statement

The datasets presented in this study can be found in online repositories. The RNA sequencing data presented in the study are deposited in the Japanese Genotype-phenotype Archive (https://www.ddbj.nig.ac.jp/jga/), accession number JGAD000750. Count data for 60230 genes are provided as Table S4 in the article/Supplementary material.

## Ethics statement

The studies involving human participants were reviewed and approved by Juntendo University Faculty of Medicine, Sugiyama Clinic, The National Center for Child Health and Development. The patients/participants provided their written informed consent to participate in this study.

## Author contributions

KK and KO conceived the project. MK, RS, KO, and KK contributed to the endometrial material. KO, JT, and KN obtained and analyzed the data. KH and AI supervised the study. KO and JT prepared the initial manuscript draft. KN and JT reviewed and finalized the manuscript. All authors have read and approved the final manuscript.

## Funding

This study was supported by the Practical Research Project for Rare/Intractable Diseases Program of the Japan Agency for Medical Research and Development (AMED) (grant number 22ek0109489h0003 to KN and KH).

## Conflict of interest

The authors declare that the research was conducted in the absence of any commercial or financial relationships that could be construed as a potential conflict of interest.

## Publisher’s note

All claims expressed in this article are solely those of the authors and do not necessarily represent those of their affiliated organizations, or those of the publisher, the editors and the reviewers. Any product that may be evaluated in this article, or claim that may be made by its manufacturer, is not guaranteed or endorsed by the publisher.

## Footnotes

^1^ https://www.proteinatlas.org/
