# Supplementary material for: Gene expression signatures associated with chronic endometritis revealed by RNA sequencing
Source: Front Med (Lausanne). 2023 Jul 20;10:1185284. doi: 10.3389/fmed.2023.1185284 (PMC10400718; doi:10.3389/fmed.2023.1185284)
Supplement: Supplementary file 1 [file Data_Sheet_1.ZIP › supplemental_materials_Oshina_et_al/SupplementaryData1_Oshina_et_al.pptx]

## Slide 1
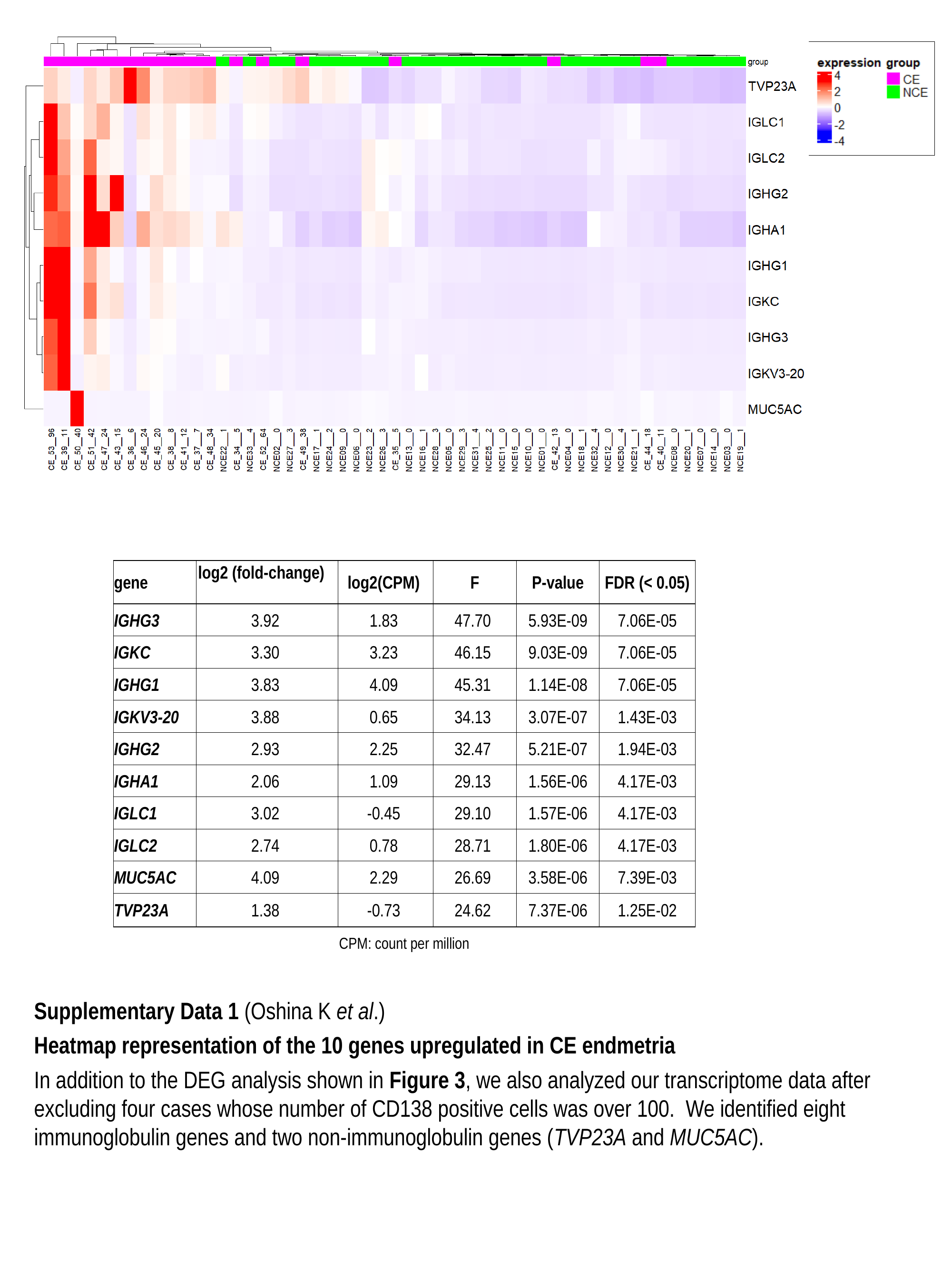

| | | | | | | | |
| --- | --- | --- | --- | --- | --- | --- | --- |
| | gene | log2 (fold-change) | log2(CPM) | F | P-value | FDR (< 0.05) | |
| | IGHG3 | 3.92 | 1.83 | 47.70 | 5.93E-09 | 7.06E-05 | |
| | IGKC | 3.30 | 3.23 | 46.15 | 9.03E-09 | 7.06E-05 | |
| | IGHG1 | 3.83 | 4.09 | 45.31 | 1.14E-08 | 7.06E-05 | |
| | IGKV3-20 | 3.88 | 0.65 | 34.13 | 3.07E-07 | 1.43E-03 | |
| | IGHG2 | 2.93 | 2.25 | 32.47 | 5.21E-07 | 1.94E-03 | |
| | IGHA1 | 2.06 | 1.09 | 29.13 | 1.56E-06 | 4.17E-03 | |
| | IGLC1 | 3.02 | -0.45 | 29.10 | 1.57E-06 | 4.17E-03 | |
| | IGLC2 | 2.74 | 0.78 | 28.71 | 1.80E-06 | 4.17E-03 | |
| | MUC5AC | 4.09 | 2.29 | 26.69 | 3.58E-06 | 7.39E-03 | |
| | TVP23A | 1.38 | -0.73 | 24.62 | 7.37E-06 | 1.25E-02 | |
| | | | CPM: count per million | | | | |
Supplementary Data 1 (Oshina K et al.)
Heatmap representation of the 10 genes upregulated in CE endmetria
In addition to the DEG analysis shown in Figure 3, we also analyzed our transcriptome data after excluding four cases whose number of CD138 positive cells was over 100. We identified eight immunoglobulin genes and two non-immunoglobulin genes (TVP23A and MUC5AC).
